# Connections between *Klebsiella pneumoniae* bloodstream dynamics and serotype-independent capsule properties

**DOI:** 10.1128/iai.00641-25

**Published:** 2026-01-29

**Authors:** Emily L. Kinney, Drew J. Stark, Saroj Khadka, Christine M. Tin, Timothy W. Hand, William Bain, Laura A. Mike

**Affiliations:** 1 Department of Medicine, Division of Infectious Diseases, University of Pittsburgh 199716, Pittsburgh, Pennsylvania, USA; 2 Department of Immunology, University of Pittsburgh 215866, Pittsburgh, Pennsylvania, USA; 3 Department of Pediatrics, University of Pittsburgh 209879, Pittsburgh, Pennsylvania, USA; 4 UPMC Children’s Hospital of Pittsburgh, Pittsburgh, Pennsylvania, USA; 5 Institute of Infection, Immunity, and Inflammation in Children, UPMC Children’s Hospital, Pittsburgh, Pennsylvania, USA; 6 Department of Medicine, Division of Pulmonary, Allergy, Critical Care, and Sleep Medicine, University of Pittsburgh 6614, Pittsburgh, Pennsylvania, USA; 7 Veterans Affairs Pittsburgh Health System, Pittsburgh, Pennsylvania, USA; University of California Davis, Davis, California, USA

**Keywords:** *Klebsiella pneumoniae*, bacteremia, capsule, bloodstream infections, mucoidy, hmv, host-pathogen, siderophores, complement resistance, macrophages

## Abstract

*Klebsiella pneumoniae* bacteremia is a significant public health burden with a 26% mortality rate, which increases when the infecting isolate is multidrug resistant. An important virulence factor of *K. pneumoniae* is its capsule, the protective polysaccharide coat that surrounds the outer membrane and is made up of individual capsular polysaccharide (CPS) chains. The capsule can differ in composition, abundance, surface attachment, and length of the individual CPS chains. Long, uniform CPS chains are associated with a high level of mucoidy. Typically, mucoidy is produced by the hypervirulent *K. pneumoniae* (hvKp) pathotype, which is associated with invasive community-acquired infections. In contrast, the classical *K. pneumoniae* (cKp) pathotype tends to be less mucoid or non-mucoid and is associated with nosocomial infections and multidrug resistance. There are over 80 serotypes of *K. pneumoniae* capsule. Capsule swap experiments have begun to reveal the effect of serotype on virulence and immune interactions. Clinically, the K2 capsule serotype is a common serotype associated with neonatal bloodstream infections. Both cKp and hvKp can produce K2 capsule, but how K2-encoding cKp and hvKp strains differ in a bloodstream infection remains unknown. To fill this gap in knowledge, we characterized the surface properties of K2 serotype cKp and hvKp bloodstream infection isolates then tested the fitness of these strains in bloodstream infection-related *in vitro* and *in vivo* assays. Understanding how K2 cKp and hvKp strains differ in pathogenic potential provides further insights into how *K. pneumoniae* capsule properties influence bloodstream infection pathogenesis.

## INTRODUCTION

Globally, *Klebsiella pneumoniae* is the third leading cause of antimicrobial resistance-related deaths (1). It is a gram-negative pathogen that infects a variety of host sites, including the urinary tract, lungs, and blood*. K. pneumoniae* bacteremia has a 26% mortality rate, which increases with antimicrobial resistance (2). According to the Child Health and Mortality Prevention Surveillance network, *K. pneumoniae* is implicated in 1 in 4 deaths among children under age 2 in low- and middle-income countries (3–5). It is also the primary cause of neonatal sepsis (6–8). There are two distinctly alarming pathotypes of *K. pneumoniae,* classical (cKp) and hypervirulent (hvKp). cKp strains are generally associated with nosocomial infections and multidrug resistance (9, 10). Meanwhile, hvKp strains are associated with community-acquired and invasive infections (11). Despite these observed pathogenic differences, both pathotypes can encode similar virulence properties.

The capsule is the protective polysaccharide coat that surrounds the outer membrane of *K. pneumoniae* and is composed of individual capsular polysaccharide (CPS) chains. The CPS can differ in serotype (capsule composition), abundance (total capsule synthesized), chain length (mode length of individual CPS chains), chain length diversity (range of CPS chain length), and attachment (outer membrane association). These nuanced capsular properties contribute to different physiological and pathogenic functions in bacteria (12). Compared to cKp, hvKp has been reported to exhibit increased capsule abundance, mucoidy, and other virulence factors linked with increased frequency of invasive infections (10, 13, 14). Recent work demonstrated that mucoidy is not driven by increased capsule abundance; rather, mucoidy is due to increased CPS chain length uniformity (15–17). While the presence of the capsule blocks complement-mediated killing, mucoidy blocks macrophage association (18–21).

There are over 80 different *K. pneumoniae* capsule serotypes, all with a different composition. However, most of the K-serotype diversity is observed in cKp as hvKp primarily produces K1 and K2 (14, 22–27). In recent years, elegant capsule swap experiments have begun to reveal the effect of individual serotypes on virulence, immune interactions, and phage dynamics (28–30). In a recent study by Huang et al., swapping the K2 capsule biosynthesis locus with either the K1 locus or a complemented K2 locus caused 100% mortality in mice, and all intravenous infections had high bacteremia (28). When the K2 capsule was replaced with the K3 or K23 serotype, the mice exhibited 100% survival, and intravenous infections had low bacteremia. Their study highlights the importance of considering the capsule serotype in *K. pneumoniae* studies. More broadly, studies comparing cKp and hvKp strains have typically focused on antibiotic resistance profiles, mucoid phenotype, or overall virulence (31–33). However, most of these comparisons do not control for capsule serotype. Since capsule serotype can significantly affect the outcome of an infection, including different serotypes when comparing cKp and hvKp could confound data interpretation (34). To overcome this limitation, we have compared virulence phenotypes of K2 serotype cKp and hvKp isolates since the K2 capsule serotype is prevalent in neonatal sepsis and community-acquired and hospital-acquired infections (6, 35, 36).

Here, we aimed to understand how K2 cKp and hvKp differ in their interaction with host defense mechanisms in the context of bloodstream infections. To assess the role of serotype-independent capsule properties in bacteremia, we studied nine K2 bloodstream isolates (cKp *N* = 5; hvKp *N* = 3) and one common K2 hvKp lab strain (ATCC 43816-derived, KPPR1) (Table 1). We quantified their capsule properties and fitness in conditions that mimic facets of bloodstream infection, specifically human serum survival, whole blood survival, host cell association, and dissemination from the bloodstream. Surprisingly, K2 cKp and K2 hvKp exhibited similar phenotypes in many assays. However, hvKp strains tend to be more mucoid, associate less with host cells, and achieve higher bacterial burdens in murine spleens and livers compared to cKp strains. These studies begin to reveal the serotype-independent role of specific capsule properties in a bloodstream infection.

**TABLE 1:** Summary of K2 isolate genotypic properties^*a*^^,^^*b*^

Isolate	Reference	Pathotype	Sequence type	Life identification number (LIN) code	*wzi* Type	K Type	*rmp* locus	*rmpA2* locus	*peg-344*	Siderophore loci			Predicted virulence score	O locus	Predicted O type
										*ybt* locus	*iro* locus	*iuc* locus			
cKp83	(37)	cKp	25	0_0_388_1_49_0_0_0_0_0	*wzi72*	K2	–	*–*	*–*	*ybt14*	–	–	1	O1/O2v2	O1ab
KLP203	(38)	cKp	25	0_0_388_0_1_0_9_0_1_0	*wzi72*	K2	–	*–*	*–*	*ybt14*	–	–	1	O1/O2v2	O1ab
KLP679	(38)	cKp	25	0_0_388_0_1_0_9_0_28_0	*wzi72*	K2	–	*–*	*–*	*ybt14*	–	–	1	O1/O2v2	O2afg
Kp6984	(39)	cKp	14	0_0_1_1_71_0_0_0_0_0	*wzi2*	K2	–	–	–	–	–	–	0	O1/O2v1	O1ab
Kp11996	(39)	cKp	14	0_0_1_1_26_2_5_0_0_0	*wzi2*	K2	–	–	–	–	–	–	0	O1/O2v1	O1ab
KPPR1	(40–43)	hvKp	493	0_0_209_1_0_0_0_0_0_0	*wzi2*	K2	*rmp3*	*–*	Present	*ybt2*	*iro3* (truncated)	–	1	O1/O2v1	O1ab
KPN165	(44)	Putative hvKp	380	0_0_339_0_16_0_0_0_0_0	*wzi203*	K2	*rmp2*	–	Present	ybt14 (truncated)	iro2 (truncated)	*iuc2*	4	O1/O2v1	O1ab
hvKp1	(37, 45)	hvKp	86	0_0_395_0_13_1_0_0_0_0	*wzi2*	K2	*rmp1*	*rmpA2_6-60%*	Present	*ybt1-*	*iro1*	*iuc1*	4	O1/O2v1	O1ab
KPN49	(44)	hvKp	66	0_0_278_0_1_4_0_0_0_0	*wzi257*	K2	*rmp2*	*–*	Present	*ybt12*	*iro2*	*iuc2*	5	O1/O2v2	O1ab

^*a*^ Pathogenwatch was used to predict the listed properties based on whole-genome sequencing data (46–49). The BIGSdb-Pasteur database was used to determine the life identification number (LIN) of the isolates (50, 51).

^*b*^ –, the absence of a gene.

## RESULTS

### K2 cKp and hvKp differ in mucoidy, not capsule abundance

Nine K2 bloodstream isolates and one common K2 hvKp lab strain (ATCC 43816-derived, KPPR1) were selected to investigate the relationship between serotype-independent capsule properties and bloodstream infection dynamics (Table 1). Based on whole-genome sequencing, all strains had at least two allele mismatches in the core genome and a range of differences in auxiliary genes, indicating that each is a unique strain (Table 1) (50). Strains were classified into pathotype based on the presence of the hypervirulent biomarkers: *rmp*, *rmpA2*, *peg-344*, *iuc*, and *iro* (37, 52). Since these genes are usually carried together on a hvKp plasmid, the presence of these genes predicts the presence of the hvKp plasmid (37). None of the cKp isolates had any of these biomarkers (Table 1). While hvKp1 had all five hypervirulent biomarkers, KPPR1, KPN165, and KPN49 had 3–4 of these hypervirulent biomarkers. Murine mortality from other studies has provided evidence that KPPR1, KPN49, and hvKp1 are hvKp (40, 53, 54). Thus, we designate KPN165 as a putative hvKp strain due to its lack of all five biomarkers and murine fatality data. KPPR1 has a low predicted virulence score due to the absence of aerobactin, but all other hvKp and putative hvKp strains had virulence scores of 4–5, and all cKp strains had virulence scores of 0–1 (Table 1). Based on these analyses, we will henceforth collectively refer to KPPR1, KPN165, KPN49, and hvKp1 as hvKp strains, even though KPN165 is missing one hvKp biomarker and supporting LD_50_ data.

**Fig 1:**
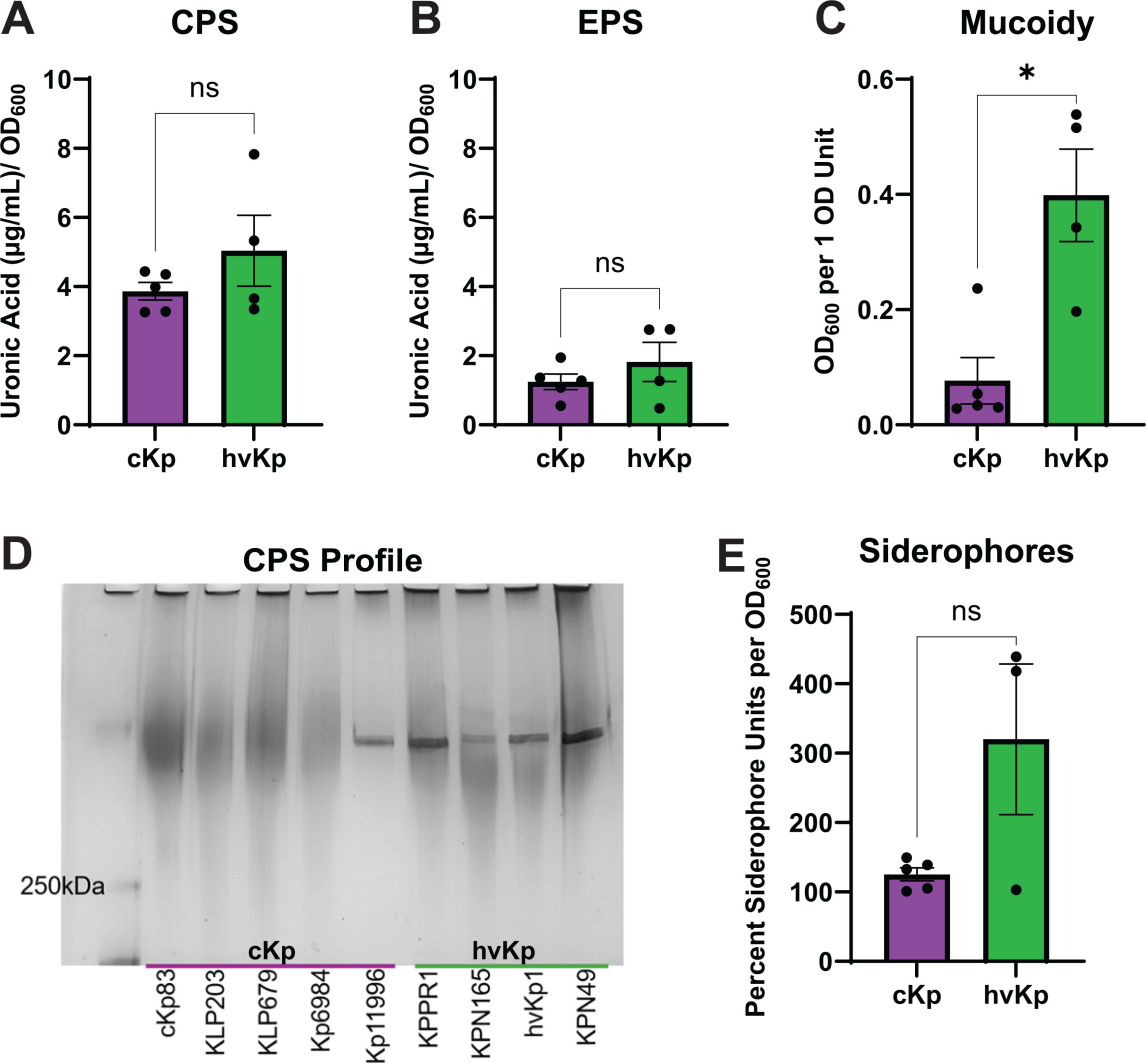
K2 cKp and hvKp strains differ in mucoidy, not capsule abundance. Capsule characteristics and siderophore quantity of K2 cKp and hvKp strains were determined. Uronic acid quantification measured (**A**) cell-associated CPS and (**B**) cell-free EPS abundance. (**C**) Sedimentation resistance was used to measure mucoidy. (**D**) Purified total CPS was resolved by a SDS-PAGE gel to visualize the CPS chain length distribution. Shown is a representative image from ≥3 independent experiments. (**E**) The colorimetric CAS assay was used to quantify siderophores. KPPR1 was excluded since it does not produce aerobactin. (**A–C, E**) Each data point represents the average of a single strain, where data were collected in triplicate ≥3 independent times. Individual data points for each strain are plotted in Fig. S1. Bars represent the mean, and error bars represent the standard error of the mean. To determine statistical significance, a normality test was first applied. A Mann-Whitney test was used in A, and an unpaired *t*-test was used in B, C, and E, where *P < 0.05 and ns = not significant.

hvKp strains are canonically described as exhibiting increased levels of capsule, mucoidy, and siderophores compared to cKp strains. To test whether this distinction holds while controlling for the capsule serotype, we tested the bloodstream infection isolates (Table 1) for cell-associated CPS and cell-free extracellular polysaccharide (EPS) production (55). Unexpectedly, both cKp and hvKp isolates produce similar quantities of CPS and EPS (Fig. 1A and B). Although mean CPS abundance was the same between the pathotypes, the hvKp strain KPN49 produced more CPS than each cKp strain (Fig. S1A). We then quantified mucoidy using low-speed centrifugation (55). As expected, the cKp isolates display less mucoidy than hvKp (Fig. 1C). Notably, we also observed that one cKp strain, Kp11996, was as mucoid as hvKp strain KPN165 (Fig. S1C). Finally, the cell-associated CPS chain length distribution was examined by resolving CPS on a SDS-PAGE gel and visualizing the polysaccharides with Alcian blue followed by a silver stain (55). Prior work has shown that strains with high mucoidy produce a uniform band of CPS, while non-mucoid strains produce a diverse smear of CPS (15, 21, 56). All hvKp strains and the mucoid cKp strain, Kp11996, produce a uniform CPS band, whereas all the non-mucoid cKp strains produce a diverse CPS smear (Fig. 1D). Notably, the mucoid level quantified by a sedimentation assay predicted CPS chain length uniformity by SDS-PAGE, even for cKp strain Kp11996 (Fig. S1C; Fig. 1D). In addition to capsule characteristics, we quantified the amount of siderophores produced by each of the strains. Since the lab strain KPPR1 does not produce aerobactin, which accounts for the majority of siderophore production and is a defining feature of hvKp, this strain was excluded from the pathotype comparison for siderophore production (37, 57, 58). Likely due to the small number of strains, there was a numerically but not statistically significant difference in siderophore production between the two pathotypes (Fig. 1E). While KPN49 had similar levels of siderophores to the classical strains, hvKp1 and KPN165 produced 3.3-fold more siderophores than the classical strains (Fig. S1D).

### K2 cKp and hvKp exhibit similar growth kinetics

**Fig 2:**
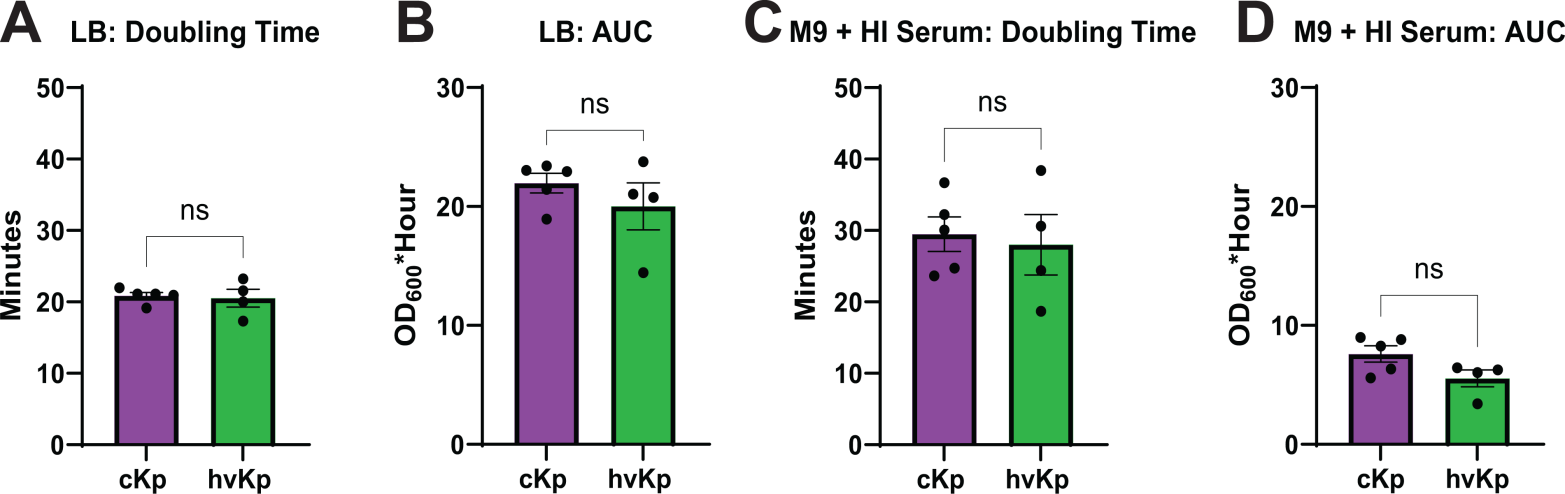
K2 cKp and hvKp exhibit similar growth properties. K2 cKp and hvKp strains were cultured in (**A, B**) LB or (**C, D**) M9 medium with 20% HI Serum as the carbon source. Growth was monitored by OD_600_ for 16 h. The (**A, C**) doubling time and (**B, D**) AUC were calculated from each growth curve. Each data point represents the average of a single strain, where data were collected in triplicate ≥3 independent times. Full growth curves are provided in Fig. S2, and individual data points for each strain are plotted in Fig. S3. Bars represent the mean, and error bars represent the standard error of the mean. To determine statistical significance, first, a test of normality was used. An unpaired *t*-test was used for A, B, and C, and a Mann-Whitney test was used for D, where ns = not significant.

To determine if growth properties could shape differences in cKp and hvKp pathogenesis, we assessed strain growth in two different media. Strains were cultured in LB or M9 minimal medium with 20% heat-inactivated serum (HI Serum) as the carbon source, and the OD_600_ was measured every 15 min to generate a growth curve (Fig. S2). The doubling time and area under the curve (AUC) were calculated from each growth curve to quantify the maximal growth rate and yield (Fig. S3). No significant differences between cKp and hvKp were detected in doubling time or AUC in either condition (Fig. 2). Therefore, any observed pathogenic differences are not likely due to growth differences.

### K2 cKp and hvKp have comparable human serum resistance, but cKp has greater C3 deposition

**Fig 3:**
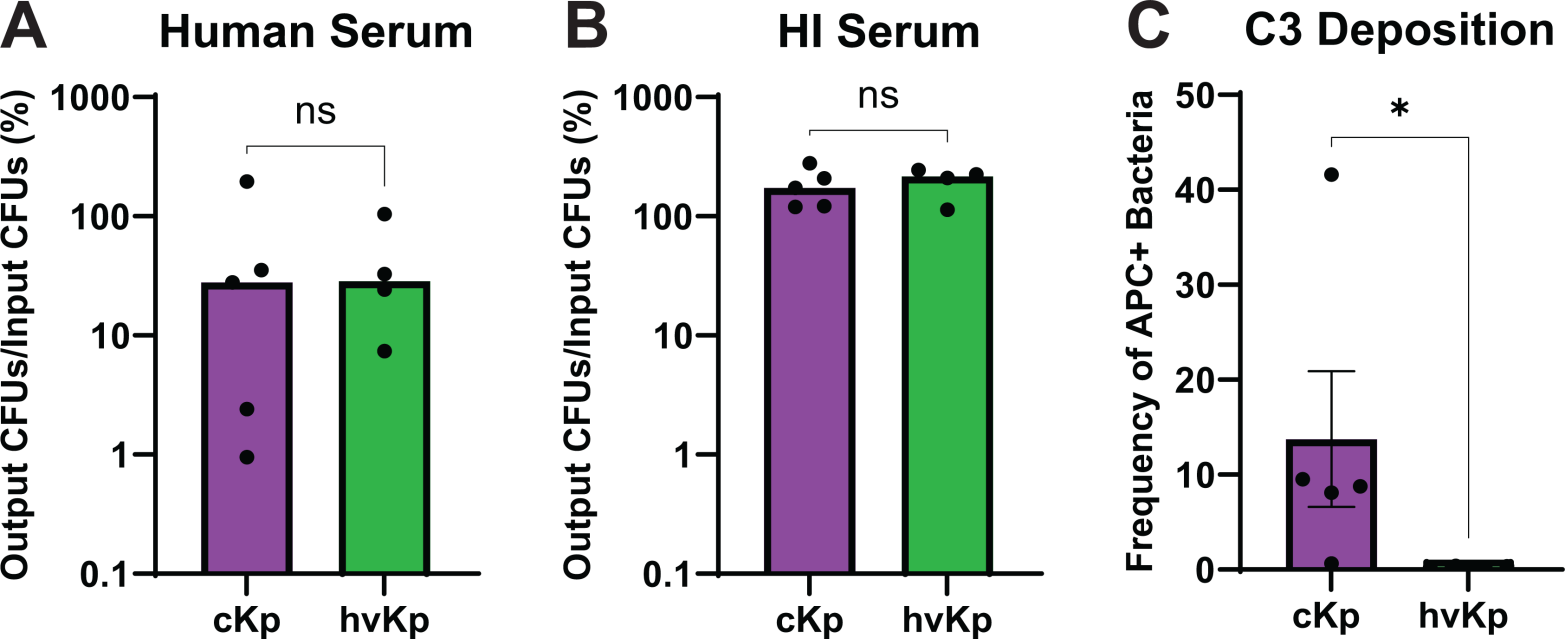
K2 cKp and hvKp have comparable human serum resistance, but cKp have greater C3 deposition. (**A**) K2 cKp and hvKp were incubated in 90% pooled human serum for 90 min. (**B**) HI serum was used as a control. Data are presented as the percentage of output colony-forming units (CFUs) divided by the input CFUs. Each data point represents the average of a single strain, where data were collected in triplicate ≥3 independent times. Each bar represents the median. Data for individual strains are presented in Fig. S4. (**C**) Strains were incubated with 10% human serum for 30 min and stained with SYTO BC green nucleic acid stain and C3b/iC3b-APC. The percentage of the bacterial population with APC stain is shown, where each data point represents the average of a single strain, collected ≥2 independent times. Each bar identifies the mean, and error bars represent the standard error of the mean. Individual APC histograms are shown in Fig. S5. To determine statistical significance, an unpaired *t*-test was used for A and B and a Mann-Whitney test for C, where ns = not significant and *P < 0.05.

An important part of the immune system in the bloodstream is the complement cascade. In *Streptococcus pneumoniae*, the capsule serotype is a factor in complement resistance (59). Since our strains are all K2, we assessed whether there was a significant difference between cKp and hvKp sensitivity to pooled human serum when capsule serotype is not a variable. cKp and hvKp survive similarly in human serum, but cKp exhibit a bimodal-like distribution (Fig. 3A). No killing was observed in HI Serum, which served as a control to confirm bacterial killing was complement-mediated (Fig. 3B). The data for the individual strains are shown in Fig. S4. Next, we considered whether complement deposition predicted serum resistance. Cleavage of complement component C3 leads to C3b deposition on the bacteria surface as an important precursor to the pathway ending in MAC formation. We measured C3b deposition using flow cytometry. Interestingly, despite no difference in complement resistance between the pathotypes (Fig. 3A), we observed higher C3b deposition on K2 cKp strains compared to the K2 hvKp strains (Fig. 3C). Histograms of C3b/iC3b-allophycocyanin (APC) staining are shown in Fig. S5.

### Survival of K2 cKp and hvKp is similar in human blood, but cKp associate with macrophages more than hvKp

**Fig 4:**
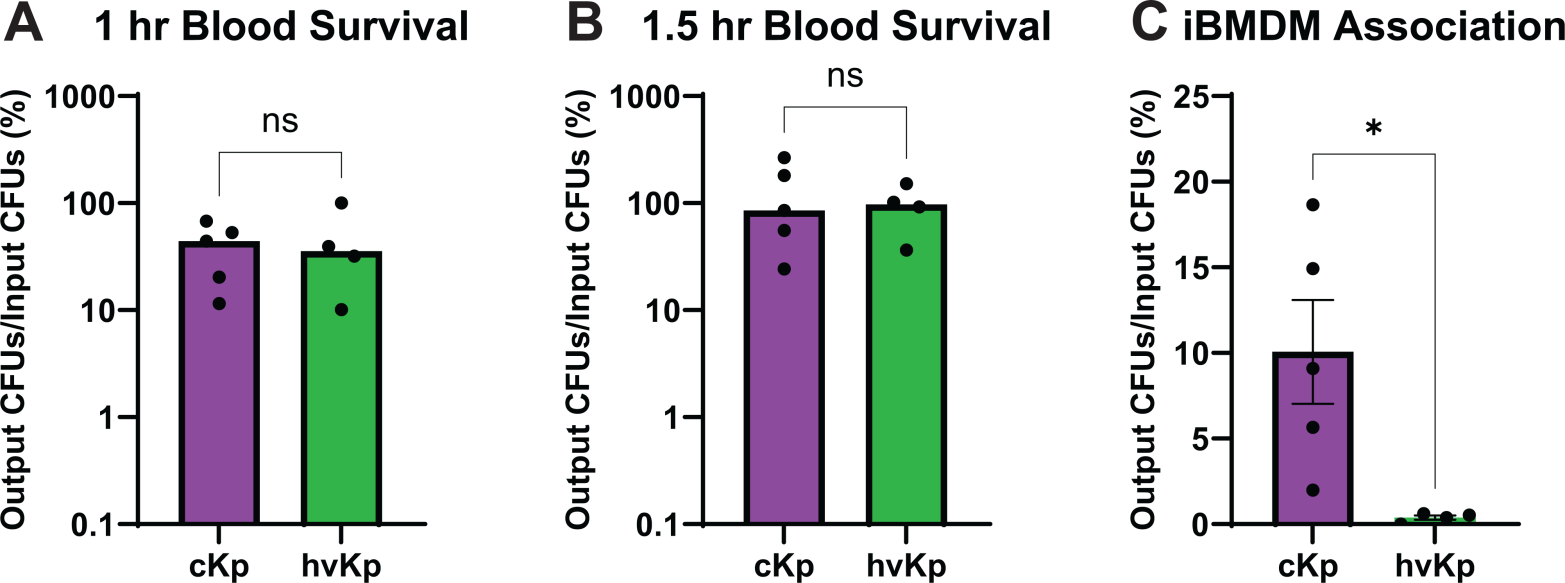
K2 cKp and hvKp survive similarly in human blood, but cKp associate with macrophages more than hvKp. K2 cKp and hvKp were incubated in 90% fresh whole human blood for (**A, B**) 60 or 90 min. (**C**) Strains were incubated with immortalized bone marrow-derived macrophages (iBMDMs) at a MOI of 10 for 2 h and then washed extensively prior to plating whole iBMDM lysates. For all graphs, data are presented as a percentage of the output CFUs divided by the input CFUs. Each data point represents the average of a single strain, where data were collected in triplicate ≥3 independent times. For A and B, each bar identifies the median. For C, the bar represents the mean, and the error bars represent the standard error of the mean. To determine statistical significance, an unpaired *t*-test (**A,B**) or Mann-Whitney test was used (**C**), where ns = not significant and *P < 0.05.

Given the lack of differences in human serum survival, we next assessed bacterial survival in whole human blood to consider whether another host factor in whole blood could influence cKp versus hvKp survival in the bloodstream. cKp and hvKp strains were incubated with human blood for either 1 or 1.5 h to compare survival patterns of the two pathotypes. cKp and hvKp exhibited similar survival at 1 or 1.5 h (Fig. 4A and B). However, there was a non-significant increase in bacteria between the two time points, indicating that after an initial period of bacterial death, *K. pneumoniae* can grow in human blood (Fig. 4A and B). Despite some donor-dependent trends in strain survival, no strain consistently survived better than other strains when data from the four donors were combined (Fig. S6A and B). Thus, cKp and hvKp survive comparably in blood.

To determine whether these pathotypes might differ in their interactions with host immune cells, we measured K2 cKp and hvKp association with iBMDMs. Due to antibiotic-resistance profiles, a gentamicin-protection assay could not be performed. Thus, the assay results include both intracellular and cell-bound bacteria. The K2 cKp isolates had a significantly higher association (26.3-fold) with the macrophages compared to the K2 hvKp isolates (Fig. 4C). Two cKp isolates, cKp83 and KLP203, had a significantly higher association than every hvKp isolate (Fig. S6C). This difference in macrophage binding suggests that reduced association of hvKp strains could contribute to the increased invasiveness of hvKp strains.

### K2 hvKp achieve higher bacterial spleen and liver burdens compared to cKp in a murine bloodstream infection model

**Fig 5:**
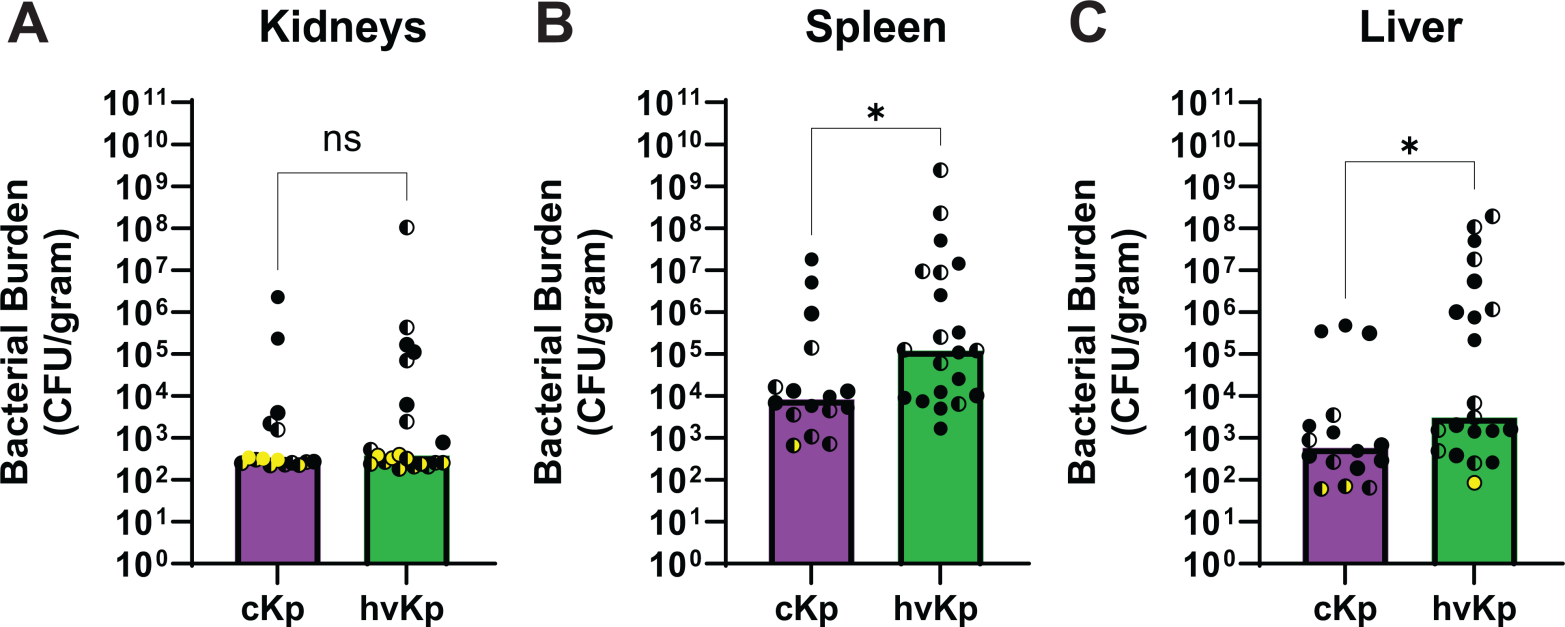
K2 hvKp achieve higher bacterial spleen and liver burdens compared to cKp in a murine bloodstream infection model. C57BL/6 mice were infected with 10^7^ CFU of cKp (Kp6984 or cKp83) or hvKp (KPN165 or hvKp1) strains. After 24 h, mice were humanely euthanized, and bacterial burdens in the (**A**) kidneys, (**B**) livers, and (**C**) spleens were enumerated. Bars represent the median, and yellow dots represent points below the limit of detection. Each data point represents a single mouse, where half circles identify male mice and full circles identify female mice. Yellow dots represent points below the limit of detection. To determine statistical significance, a Mann-Whitney test was used, where *P < 0.05 and ns = not significant. Each strain was tested on at least 2 different days with at least 8 mice.

Since K2 strains are prevalent in neonatal sepsis cases and all strains, except KPPR1, were isolated from bloodstream infections, we compared cKp and hvKp isolate dissemination patterns using a murine model of bacteremia (6, 35). Given the more invasive nature of hvKp, we expected to observe greater dissemination of hvKp compared to cKp. Mice were infected with 10^7^ CFU of cKp (Kp6984 or cKp83) or hvKp (KPN165 or hvKp1) isolates via tail vein injection, and then blood-filtering organs were harvested after 24 h. Although no single strain achieved significantly higher tissue burdens compared to other individual strains (Fig. S6A through C), comparing combined data from both hvKp strains and both cKp strains detected significantly higher hvKp bacterial burdens in the spleen and liver (Fig. 5A through C). Additionally, while 100% of cKp-infected mice survived (*N* = 17/17) at the 24-h time point, only 80.8% of hvKp-infected mice (*N* = 21/26) survived, further supporting the enhanced virulence of hvKp strains *in vivo* (Fig. S6D). Furthermore, within the hvKp isolates, 23.5% of KPN165-infected mice did not survive compared to 11% of hvKp1-infected mice, indicating that KPN165 is as virulent or more virulent than hvKp1. Although we did not determine the LD_50_ of KPN165, these *in vivo* data support that KPN165 is likely a *bona fide* hvKp isolate. The decreased organ burden in cKp-infected mice suggests that cKp are less fit *in vivo*, or alternatively, that hvKp have an enhanced capacity for spleen and liver colonization.

### Linear regressions reveal that some bacterial properties and host factors are linked

**Fig 6:**
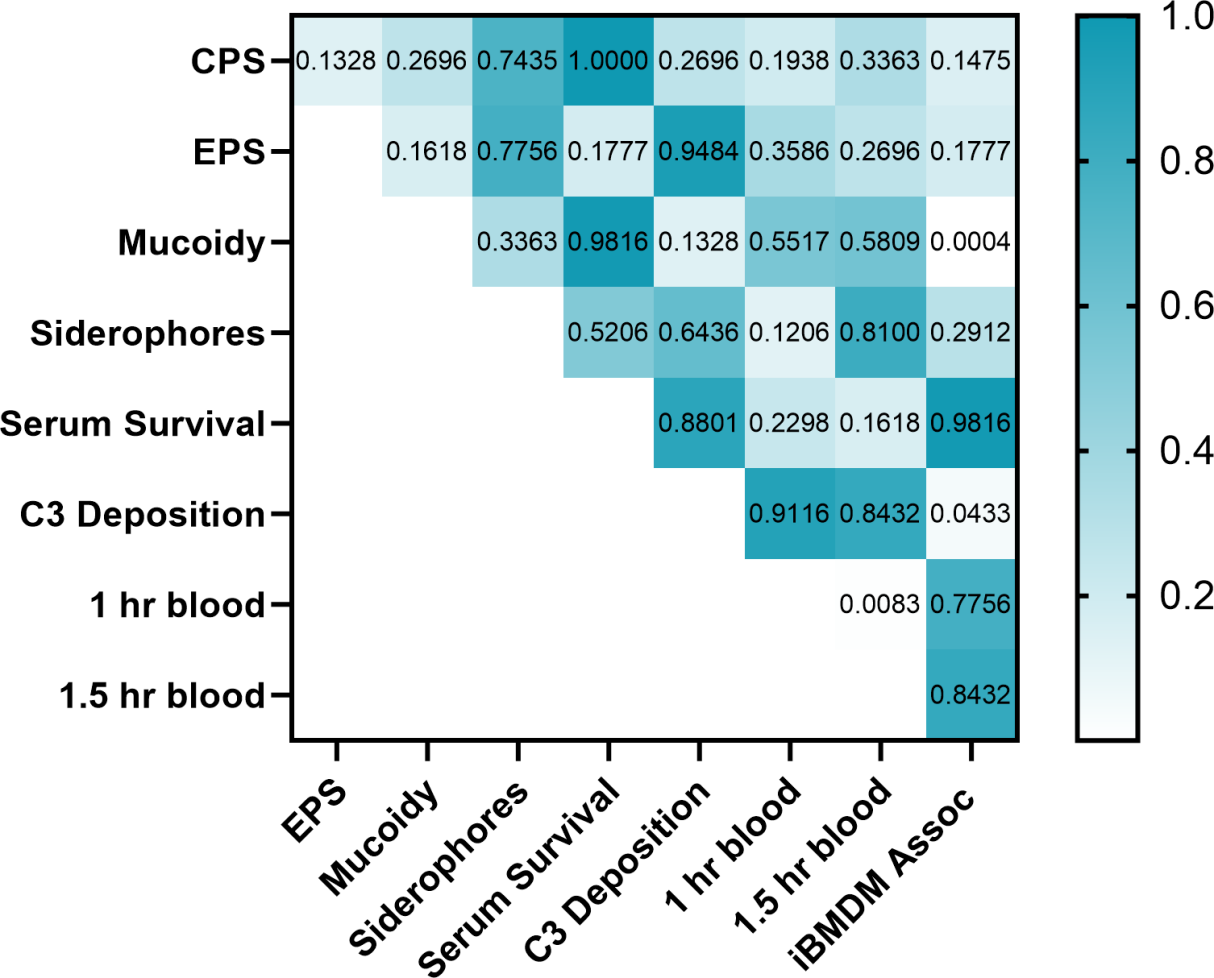
Correlations between bacterial properties and host interactions. A correlation matrix was used to compare relationships between quantified properties in Fig. 1, 3 and 4, using a nonparametric Spearman correlation and two-tailed *P-*values with a 95% CI. The *P-*values are displayed, and *P-*values below 0.05 were deemed significant.

To determine whether any bacterial properties or host factors that we assessed interacted, we performed a correlation matrix between each factor. Our correlation analyses predict linkages between mucoidy and macrophage association (*P = 0.0004*), macrophage association and C3 deposition (*P = 0.0433*), and early-blood survival and late-blood survival *(P = 0.0083*) (Fig. 6). This does not necessarily mean that these properties depend on each other; for example, these properties could share related regulatory mechanisms or partition to a certain pathotype. While we primarily focused our analyses on capsule-related features, there may be other non-capsular factors affecting these phenotypes that we did not consider. One correlation that we expected to detect was between capsule abundance and human serum survival. Since the presence of the capsule has an established role in resisting complement-mediated killing, we hypothesized that increased capsule abundance would block the complement from reaching the cell surface (18–20). However, we did not detect any correlations between human serum survival and CPS or EPS abundance. In addition, we predicted that hvKp strains would withstand complement-mediated killing better than cKp strains, which could provide one mechanism for their invasive pathogenesis, but the hvKp isolates did not resist complement-mediated killing better than the cKp isolates (Fig. 3A). Moreover, the correlation matrix revealed no significant correlation between serum resistance and any quantified capsule characteristics (CPS abundance, EPS abundance, or mucoidy) (Fig. 6**)**. Thus, when we only consider K2 serotype strains, neither the hvKp pathotype nor any capsule properties predicted serum resistance (Fig. 3 and 6). Notably, mucoidy was inversely correlated with iBMDM association, and both of these properties are significantly different between cKp and hvKp. This suggests that increased mucoidy and reduced iBMDM association could promote hvKp systemic dissemination, rather than increased CPS abundance or resistance to complement-mediated killing.

## DISCUSSION

The cKp and hvKp pathotypes have been used to categorize *K. pneumoniae* and predict the pathogenic properties of a specific strain, but the vast genomic variation cannot be understated (60). This variation makes it risky to assume phenotypic homogeneity within each pathotype. Traditionally, the invasiveness of hvKp strains was attributed to elevated capsule abundance, mucoidy, and siderophore production (10, 13, 14, 61). However, most studies comparing these pathotypes have used isolates with a variety of capsule serotypes. We now know that capsule serotype itself alters the virulence, confounding these prior studies as hvKp strains tend to express the more immune-evasive capsule serotypes (K1 and K2) (28, 62). The experiments presented here reveal extensive phenotypic overlap between the two pathotypes. However, our study is limited by the number of isolates examined. Furthermore, the genomic diversity of the clinical isolates means that we cannot pinpoint the genetic differences underlying the observed phenotypic differences. Nonetheless, these data have identified several testable hypotheses for future studies. Compared to cKp, we observed that hvKp strains are more mucoid, have increased evasion of C3 deposition, associate less with macrophages, and achieve higher spleen and liver burdens, but exhibit no significant differences in CPS or EPS abundance, siderophore quantity, growth, serum resistance, or blood survival (Fig. 1 to 5). Even if a significant phenotypic difference was detected between cKp and hvKp, there was usually at least one strain whose phenotype matched the other pathotype. For example, the mucoidy of cKp strain Kp11996 mirrored the mucoidy of hvKp pathotype strains (Fig. S1C). However, this does not mean a single phenotype predicts strain virulence. In fact, prior work has proposed that a collection of biomarkers (*iucA*, *iroB*, *peg-344*, *rmpA*, and *rmpA2*) predict pathogenic differences between cKp and hvKp (63). Even though our data indicate that we cannot predict the specific behavior of an individual strain based solely on the pathotype, by controlling for serotype, our relatively small data set clarifies some relationships between surface characteristics and host interactions.

Our host assays tested bacterial interactions with cells and soluble bloodstream factors as phagocyte and complement activity are well-established immune components during *K. pneumoniae* bacteremia (64–66). We used pooled human serum to examine complement resistance and whole human blood to consider other soluble factors in blood that may control bacterial growth (67). The whole human blood assay captured both bacterial killing (1 hr) and bacterial growth (1.5 hr) (Fig. 4A and B). This allows us to hypothesize that while blood components initially control *K. pneumoniae* growth, some bacteria survive and can use blood as a nutrient source once immune components are depleted *ex vivo*. While the two pathotypes did not exhibit differences in human serum or human blood survival, hvKp had lower C3 deposition compared to cKp (Fig. 3 and 4; Fig. S5). This suggests that hvKp may evade complement-mediated killing by blocking C3 activation or deposition, while complement-resistant cKp may use an alternative mechanism. Thus, cKp and hvKp may employ different mechanisms to resist complement-mediated lysis, independent of the serotype. Since we did not see differences in complement-mediated killing between the pathotypes or any correlations linking capsule characteristics with resistance to complement-mediated killing, we began investigating interactions with phagocytes.

To examine how these two pathotypes differ in phagocyte interactions, we measured cKp and hvKp binding to iBMDMs, an important precursor to phagocytosis. Phagocytosis was not measured here because the standard measure of phagocytosis is a gentamicin-protection assay, and some isolates are gentamicin-resistant. A previous study showed that serotype controls the ability of liver-resident macrophages (Kupffer cells) to capture *K. pneumoniae* (28). Since macrophages are an established line of defense against *K. pneumoniae* bloodstream infections, we used iBMDMs to model bacteria-host cell association (64, 68, 69). cKp exhibited a higher association with iBMDMs compared to hvKp, which inversely correlated with mucoidy, suggesting that mucoidy could shape bacterial-host immune cell binding (Fig. 4C and 6). This agrees with previous reports that hypermucoid strains associate less with macrophages and epithelial cells (16, 17, 70). Although the bacteria were not opsonized in the iBMDM association assay, opsonization increases cellular control of *K. pneumoniae* infections (71–73). The lack of C3b deposition on hvKp strains could further enhance the evasion of macrophage phagocytosis, increasing hvKp *in vivo* fitness. Additionally, C3b deposition and iBMDM association were correlated, suggesting that directly or indirectly related mechanisms may block bacterial interactions with these host elements. These results altogether led us to conclude that how cKp and hvKp interact with macrophages is a critical difference between the pathotypes. However, these phenotypes and their correlation could be due to non-capsular factors since the virulence properties are linked on the hvKp virulence plasmid (37). Further studies are needed to confirm the role of capsule properties in evading C3b deposition and iBMDM association.

Although the *ex vivo* assays provide some insights into how differences between K2 cKp and hvKp affect host interactions, we also wanted to examine the effect of pathotype differences during bloodstream dissemination. We selected two representative strains from each pathotype to minimize animal use. Within each pathotype, the selected strains have different levels of complement resistance and did not share the sequence type. All four strains have the same amount of CPS and EPS abundance, allowing us to remove that variable. We measured dissemination from the blood to the spleen, liver, and kidneys in a murine model. We selected these organs because they filter a large volume of blood. We observed inflammatory foci on the livers, likely abscesses, in both cKp- and hvKp-infected mice (Fig. S6E). Since this occurred in all strains, this suggests that the immune response to both pathotypes is similar in the liver. However, the two hvKp isolates had higher bacterial burdens in the spleen and liver than the two cKp isolates (Fig. 5). This suggests that the hvKp strains have a higher capacity to disseminate from the bloodstream than cKp, despite both pathotypes eliciting inflammatory foci in the livers. Alternatively, these data could indicate that the hvKp strains have increased capacity for growth or immune evasion in these sites (74). For example, low arginine abundance in the livers could optimize hvKp fitness factor expression (21). Nonetheless, from the bacterial burden and the overall survival of the mice, we can conclude that the hvKp isolates were more virulent than the cKp isolates tested in our murine model. Since the hvKp isolates used in the murine challenge both had elevated mucoidy and siderophore quantities compared to the cKp strains, we cannot conclude whether *in vivo* differences are due to mucoidy, siderophores, another factor, or a combination of these as each of these factors can contribute to hvKp *in vivo* fitness (57, 61, 75–77).

Our observation that cKp and hvKp exhibit similar survival in serum and blood, yet significant differences in iBMDM association and bacterial burden in the spleen and liver, may point to the importance of cell-mediated immunity in controlling *K. pneumoniae* bacteremia. Although complement is a major host factor in the bloodstream, our data indicate that direct serum killing may not be a major factor in the pathogenic potentials of cKp and hvKp in mice. For example, we observed that cKp isolate Kp6984, with low mucoidy and low human serum resistance, had similar bacterial organ burdens to the cKp isolate with high human serum resistance, cKp83 (Fig. S6A through C). Our results either indicate that mouse serum may not limit *K. pneumoniae* growth as effectively as human serum or that human serum may not limit *K. pneumoniae* bloodstream survival. In support of the latter conclusion, a recent case study reported that selection for resistance to phagocytosis was observed more than resistance to complement in isolates collected from the bloodstream and during thoracoabdominal repair (78). Thus, the increased invasiveness of hvKp strains could be due to differences in immune cell association, likely due to both opsonized and non-opsonized processes, rather than direct killing by soluble factors.

An additional outcome of this study is the further evidence of the importance of the capsule serotype in *K. pneumoniae* infections. The overlapping phenotypes of K2 cKp and hvKp strains bolster evidence for the importance of serotype in *K. pneumoniae* pathogenesis. This agrees with capsule swap experiments that have started to uncover the effect of serotype on virulence, immune interactions, and phage dynamics (28–30). Studies have already begun to build on these data by developing vaccines for important capsule serotypes like K2 (79–81). Additionally, our study reveals questions for follow-up studies. We have shown that strains with similar lineages have some different phenotypes, raising questions regarding the genetic features underlying these phenotypic differences and the selective pressures driving phenotype emergence. One such phenotype is the difference in resistance to complement-mediated lysis between KLP203 and KLP679, despite aligning to the eighth bin in the LIN code (Table 1; Fig. S4A). Even if this serum survival difference is due to the O type or an auxiliary gene, understanding the mechanism driving the observed difference would expand our understanding of how *K. pneumoniae* resists complement-mediated lysis.

In summary, we have revealed that K2 cKp exhibit lower mucoidy, greater macrophage association, greater C3 deposition, and lower bacterial organ burdens after dissemination from the blood compared to K2 hvKp. However, we have also shown that there is a high degree of phenotypic overlap between these two pathotypes. These data offer insights into how K2 capsule characteristics of *K. pneumoniae* impact bacteremia pathogenesis and provide further evidence for the importance of controlling for the capsule serotype in *K. pneumoniae* pathogenesis work.

## MATERIALS AND METHODS

### Strain and culture conditions

*Klebsiella pneumoniae* isolates used in this study are reported in Table 1. Unless otherwise noted, strains were cultured in low-salt LB medium or on low-salt LB agar. Liquid cultures were incubated at 37°C for 15.5–16.5 h at 200 rpm, and plates were incubated at ambient temperatures or 37°C (46). The cKp and hvKp pathotypes were determined based upon the presence of the biomarkers *rmp*, *rmpA2*, *iro*, *iuc*, and *peg-344* (63). If a strain had all 5 biomarkers or previous murine fatality data, they were labeled as hvKp. If they had 3–4 biomarkers and no supporting murine fatality data from other labs, they were labeled putative hvKp. hvKp and putative hvKp isolates were analyzed together. The murine mortality from our infections with KPN165 supports that this strain is hvKp, whereas the murine mortality from others has shown that KPPR1, hvKp1, and KPN49 are hvKp (37, 40, 53, 54).

### Uronic acid quantification

Capsule abundance was determined by uronic acid quantification, as previously described (16, 55, 82–84). To start, 250 µL of bacterial culture were added to 50 µL of either 1% Zwittergent 3-14 in 100 mM citric acid (to isolate total CPS) or distilled water (to isolate EPS). CPS samples were incubated at 50 °C for 20 min. All samples (CPS and EPS) were centrifuged at 17,000 *× g* for 5 min, and then 100 µL of the supernatant was added to 400 µL ice-cold absolute ethanol. Solutions were incubated on ice for 20 min and then centrifuged at 17,000 *× g* for 5 min. Pellets were resuspended in 200 µL of water and then incubated at 37°C for 30 min. To each sample, 1.2 mL of 0.0125 M sodium tetraborate in sulfuric acid was added and then boiled at 100°C for 5 min and cooled on ice for 5 min. The absorbance of acid-treated samples at 520 nm (A_520_) was measured. Subsequently, 10 µL of 0.3% 3-hydroxydiphenyl in 0.125 M sodium hydroxide was mixed into each sample, and the A_520_ was measured again. A glucuronic acid standard curve was used to determine the uronic acid amount in each sample. EPS was subtracted from total CPS to get cell-associated CPS. CPS and EPS levels were presented as uronic acid concentration per OD_600_.

### Sedimentation assay

Mucoidy was measured using sedimentation resistance, as in Khadka et al. (16, 55). The OD_600_ of bacterial cultures was measured and normalized to 1 OD_600_ unit in 1 mL. Then, the samples were subjected to a standard low-speed centrifugation (1,000 *× g* for 5 min), and the supernatant OD_600_ was measured. Mucoidy was plotted as the supernatant OD_600_ per 1 OD_600_ unit.

### CPS chain length visualization

To analyze the CPS chain length mode and distribution, purified CPS samples were visualized using an SDS-PAGE gel, as previously described (15, 55, 56, 85). The bacterial cultures were normalized to 1.5 OD_600_. Samples were centrifuged at 21,000 *× g* for 15 min, and then all of the supernatant, except for 50 µL (including the pellet), was removed. The remaining pellet was resuspended in 1 mL PBS. The resuspended pellet was centrifuged at 21,000 *× g* for 15 min, and then all but 250 µL was removed. A 50 µL aliquot of 1% Zwittergent 3-14 in 100 mM citric acid was added to the samples (5:1 sample:Zwittergent), followed by incubation at 50°C for 20 min. CPS samples were pelleted at 17,000 *× g* for 5 min, and then 100 µL of the supernatant was added to 400 µL ice-cold absolute ethanol. Solutions were incubated on ice for 20 min and then pelleted at 17,000 *× g* for 5 min. Pellets were rehydrated in 200 µL of water at 37°C for 30 min. Next, 75 µL of solubilized CPS samples were added to 25 µL of 4× SDS-gel loading dye, and then 20 µL of each sample was resolved on a 4%–15% Mini-PROTEAN TGX stain-free pre-cast gel (Bio-Rad) by applying 300 V for 4.5 h at 4°C. The gel was washed five times in ultra-pure water for 10 min, then stained with 0.1% Alcian blue in stain base solution (40% ethanol and 60% 20 mM sodium acetate, pH 4.75) for 1 h, and then the stain base solution was added to de-stain the gel overnight. The gel was stained using a Pierce Silver Stain Kit (ThermoFisher) and imaged on a Bio-Rad GelDoc.

### CAS assay

Siderophores were quantified using the CAS Assay (86, 87). Cultures were grown in T medium while shaking for 18 h. Cultures were centrifuged at 9,614 × *g* for 5 min, and then 500 µL of the supernatant was mixed with 500 µL CAS reagent and 10 µL of the shuttle solution (0.2 M 5-sulfosalicyclic acid). OD_630_ was measured after 5 min. Percent siderophore units were defined as [(Ar-As)/Ar]100, where Ar is the absorbance of the buffer with the CAS reagent and the shuttle solution and As is the absorbance of the sample. Percent siderophore units were divided by the OD_600_ of each sample.

T medium was made using 6.8 g/L NaCl, 3.7 g/L KCl, 1.1 g/L NH_4_Cl, 0.142 g/L Na_2_SO_4_, 0.00272 g/L KH_2_PO_4_, and 12.1 g/L tris(hydroxymethyl)aminomethane (88, 89). The pH of the solution was adjusted to 7.4. The solution was chelexed for 3 h at room temperature and then sterile-filtered. The solution was supplemented with 0.113 g/L anhydrous CaCl_2_, 0.10 g/L MgCl_2_ 6 H_2_O, 1× vitamin solution, and 0.4% glucose. One liter of 100× vitamin solution is composed of 50 mL 0.02 M thiamine HCl, 50 mL 0.02 M calcium pantothenate, 50 mL 0.02 M *p*-aminobenzoic acid, 50 mL 0.02 M *p*-hydroxybenzoic acid, and 50 mL 0.02 M 2,3-dihydroxybenzoic acid.

The CAS reagent was made by first dissolving 0.0219 g HDTMA in 50 mL water. Separately, 1.5 mL 1 mM FeCl_3_ 6 H_2_O in 10 mM HCl and 7.5 mL of 2 mM CAS solution were mixed. This solution was then slowly added to the HDTMA. In a separate beaker, 4.307 g piperazine was dissolved in 30 mL water, and then the pH was adjusted to 5.6. This solution was then added to the HDTMA/Fe/CAS solution. Then, the volume was brought to 100 mL with water.

### Bioinformatics

Pathogenwatch software (version 23.4.0 https://pathogen.watch) was used to determine the sequence type, O locus, O type, and presence of the *rmp*, *rmpA2*, *iro*, and *iuc* loci from the genomic sequence of the bacterial strains (46–49). BLAST was used to determine the presence of *peg-344*. The genomic sequences were obtained from either the NCBI public database, requested from the strain source, or sequenced on the Illumina platform (SeqCoast) (Table 2). The BIGSdb-Pasteur database was used to determine the LIN of the isolates (50, 51).

**TABLE 2:** Genome accession number

Strain	SRA or SAMN
cKp83	SAMN51531023
KLP203	SAMN10435743 SRS4051462
KLP679	SAMN38448741 SRS19728011
Kp6984	SRR18486035
Kp11996	SRR19090175
KPN165	SAMN24009745
hvKp1	Chromosome: NZ_CP152338 Plasmid: NZ_CP152339
KPN49	SAMN24009790

### Bacterial growth assay

Bacterial growth curves were generated following the protocol detailed by Pariseau et al. (46). Specifically, stationary-phase bacterial cultures were sub-cultured in LB and incubated for 1.5 h at 37°C, shaking at 200 rpm. The sub-cultured samples were diluted to 0.0001 OD_600_ in either LB or low-iron M9 minimal medium supplemented with CaCl_2_, MgSO_4_, and 20% HI human serum (Innovative Research). The samples were cultured with continuous shaking at 37°C, and the OD_600_ was measured every 15 min for 16 h using a microplate reader (Biotek Synergy HTX by Agilent).

### Human serum survival

Bacterial survival in human serum was measured as described by Mike et al. (16). Strains were centrifuged at 16,249 *× g* for 10 min and then resuspended in sterile PBS. The resuspended samples were then diluted to an OD_600_ of 0.02. The diluted bacterial preparations were incubated in 90% pooled human serum (Innovative Research) or HI serum (inactivated at 58°C for 1 h) at 37°C for 90 min. Bacterial CFUs in the input and output samples were enumerated by serial dilution and plated on LB agar. Bacterial survival was presented as the percentage of input CFUs surviving after incubation.

### C3 deposition

Complement deposition on the bacterial surface was measured using flow cytometry, as described by Bain et al. (73). Briefly, overnight bacterial cultures were sub-cultured (1:100) in LB. Once the sub-culture reached the log growth phase, the culture density was adjusted to 0.2 OD_600_ and then resuspended in GVB^o^ (ComplementTechnology #B103; no Mg or Ca). Bacteria (250 µL) were incubated with 25 µL MgEGTA and serum (Complement Tech; 50 µL normal human serum, 25 µL C3-depleted serum, or no serum) for 30 min at 37 °C. The reaction was quenched (750 µL cold, sterile PBS), pelleted (1,900 × *g* for 5 min), then washed twice by resuspending in 500 µL PBS, centrifuging, and then removing the supernatant. Each sample was resuspended in 100 µL 2% FBS in PBS, and then 50 µL was removed. Flow antibodies (2.5 µL ; 1:20 dilution per 10^6^ cells) were added to the tubes: C3b/iC3b-APC (Biolegend 846106) and SYTO BC green nucleic acid stain (Invitrogen by Thermo Fisher Scientific S34855). The reaction was incubated at 4°C for 30 min and then washed twice with PBS (centrifuged at 1,900 × *g* for 5 min, supernatant removed, then resuspended in 500 µL PBS). Samples were analyzed on a Fortessa 7 cytometer (thresholds: 200 for FSC, 200 for SSC, and 300 for SYTO BC).

### Human blood collection

Blood from healthy human donors was collected to use in the whole blood survival assay. Approximately 30 mL of blood were obtained through venipuncture after informed consent from the donor (CR19040346-008). The blood samples were collected in a sterile collection tube containing ACD-A as an anti-coagulant.

### Survival in whole blood

Overnight bacterial cultures (1 mL) were centrifuged at 21,000 *× g* for 15 min and then resuspended in 1 mL sterile PBS, followed by dilution to an OD_600_ of 0.02. Then, 10 µL of the bacterial sample was incubated in 90 µL human blood (ACD-A tube) statically at 37 °C for 60 or 90 min. Bacterial CFUs in the input and output samples were enumerated by serial dilution and plated on LB agar. Bacterial survival and/or growth were presented as the percentage of input CFUs present after incubation.

### Bone marrow-derived macrophage association assay

iBMDMs (BEI Resources NR-9456) were used to measure bacterial association to host cells (15). The macrophages were grown in a 24-well plate (*n* = 200,000 cells per well). Then, each well was infected with bacterial cells prepared in 1 mL Dulbecco’s Modified Eagle Medium (DMEM) at an MOI of 10. The infected macrophages were briefly centrifuged down (54 × *g* for 5 min) to initiate cell contact. Then, the plate was incubated for 2 h, followed by washing with 1 mL sterile PBS three times. After washing, 1 mL 0.2% Triton-X-100 was added to each well for 5 min while shaking to lyse monocytes for bacterial release. The inocula (input) and macrophage lysates (output) were serially diluted and CFUs enumerated on LB agar. Bacterial association was presented as the percentage of output CFUs relative to input CFUs.

### Murine bloodstream infection model

The *K. pneumoniae* murine bloodstream infection model used in this study was adapted from a previously described model and performed in adherence to humane animal handling recommendations and approved by the University of Pittsburgh Institutional Animal Care and Use Committee (protocol 24105753) (90). C57Bl/6 mice (7–8 weeks old) were procured from Charles River Laboratory (Ashland, OH, USA). The mice were injected with 10^7^ bacteria via tail vein. After 24 h, the mice were humanely euthanized. The liver, spleen, and kidneys were collected then homogenized in sterile PBS. Bacterial burdens were determined by serially diluting the homogenized organs and then enumerating CFUs on LB agar. Bacterial burden was presented as the log-transformed CFUs divided by organ weight.

### Statistics

All data, except for the murine bloodstream model and C3 deposition model, were collected on at least 3 different days. For these models, bacterial strains were used at least 2 different days. All statistical analyses were computed in Prism 10.3.1 (GraphPad Software, La Jolla, CA, USA). Tests of normality were performed on all data. If normal, an unpaired *t*-test was used to compare data. If not normal, a Mann-Whitney test was used. For data with more than two comparisons, a one-way ANOVA was used if the data were normal, and a Kruskal-Wallis test was used if the data were not normal. Male and female mice and human blood donors were used to consider sex as a variable.

## SUPPLEMENTAL MATERIAL

Supplemental figures iai.00641-25-s0001.pdf Fig. S1 to S7.

ASM does not own the copyrights to Supplemental Material that may be linked to, or accessed through, an article. The authors have granted ASM a non-exclusive, world-wide license to publish the Supplemental Material files. Please contact the corresponding author directly for reuse.
