# Supplementary material for: Connections between Klebsiella pneumoniae bloodstream dynamics and serotype-independent capsule properties
Source: Infect Immun. 2026 Jan 29;94(3):e00641-25. doi: 10.1128/iai.00641-25 (PMC12974125; doi:10.1128/iai.00641-25)
Supplement: Supplemental figures — Fig. S1 to S7. [file iai.00641-25-s0001.pdf]

SUPPLEMENTARY FIGURES

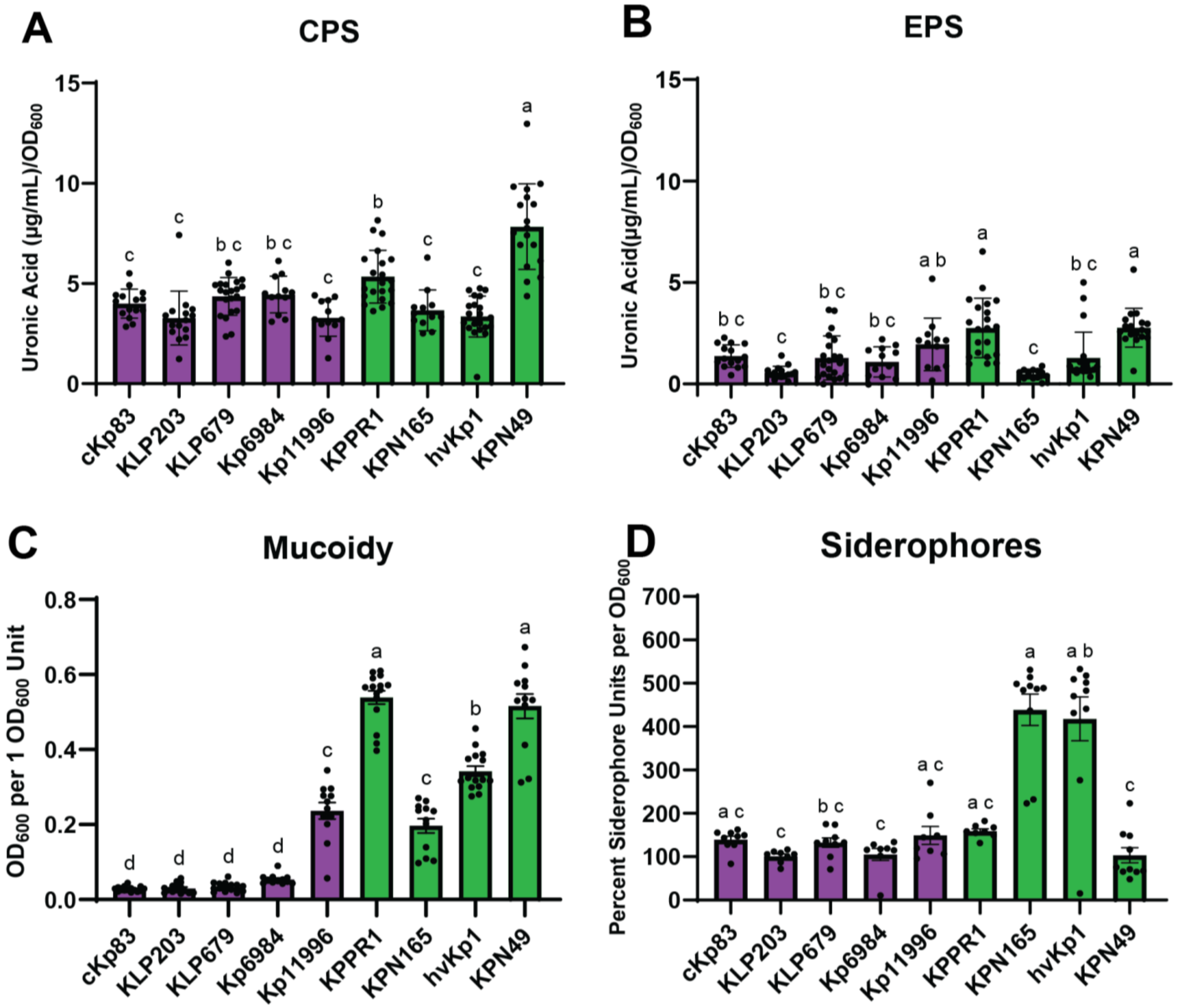

Figure S1. Capsule Characteristics of Individual Strains

Capsule characteristics of K2 cKp and hvKp strains were determined. Uronic acid quantification measured (**A**) cell-associated CPS and (**B**) cell-free EPS abundance. (**C**) Sedimentation resistance was used to measure mucoidy. (**D**) The CAS assay was used to quantify siderophores. Each bar identifies the mean, and error bars represent the standard error of the mean. A One-Way ANOVA (**A-C**) or Kruskal-Wallis test (**D**) was used to test statistical significance. Compact letter display was used to display multiple pairwise comparisons, where any two groups that share a letter have a p value greater than 0.05. Experiments were performed ≥ 3 independent times in triplicate.

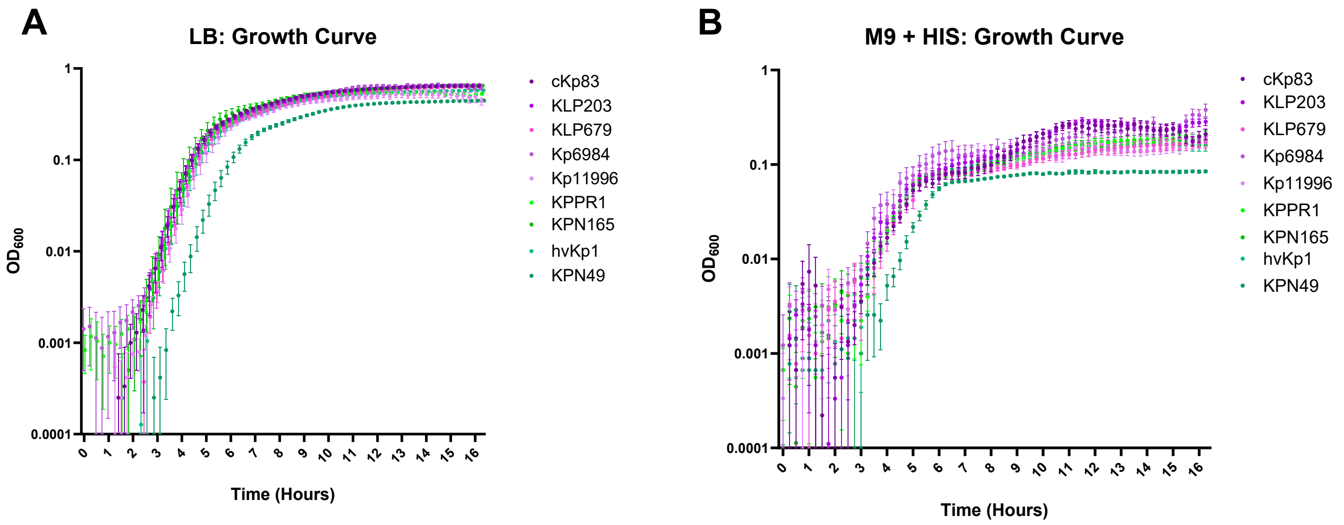

Figure S2. Growth Curves of Individual Strains

K2 cKp and hvKp strains were cultured in (**A**) LB or (**B**) M9 medium with 20% heat-inactivated serum (HIS) as the carbon source. Growth was monitored by OD_{600} for 16 hours. Each dot identifies the mean, and error bars represent the standard error of the mean.

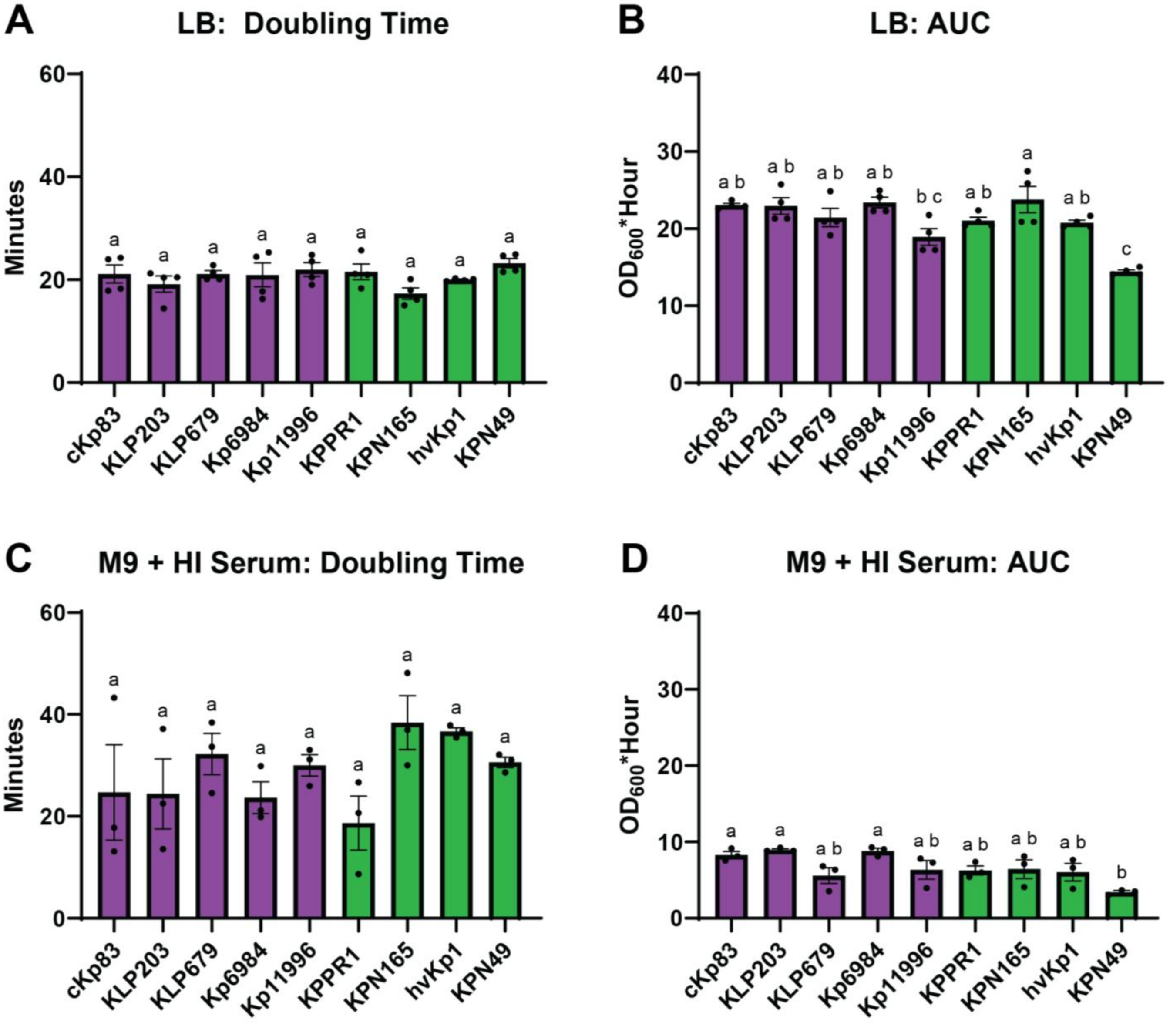

Figure S3. Growth Kinetics of Individual Strains

K2 cKp and hvKp strains were cultured in (**A, B**) LB or (**C, D**) M9 medium with 20% heat-inactivated serum (HIS) as the carbon source. Growth was monitored by OD_{600} for 16 hours. The (**A, C**) doubling time and (**B, D**) area under the curve (AUC) were calculated from each growth curve. Each bar identifies the mean, and error bars represent the standard error of the mean. To determine statistical significance, a test of normality was first used, then a one-way ANOVA was applied. Compact letter display was used to display multiple pairwise comparisons, where any two groups that share a letter have a p value greater than 0.05. Experiments were performed ≥ 3 independent times in triplicate.

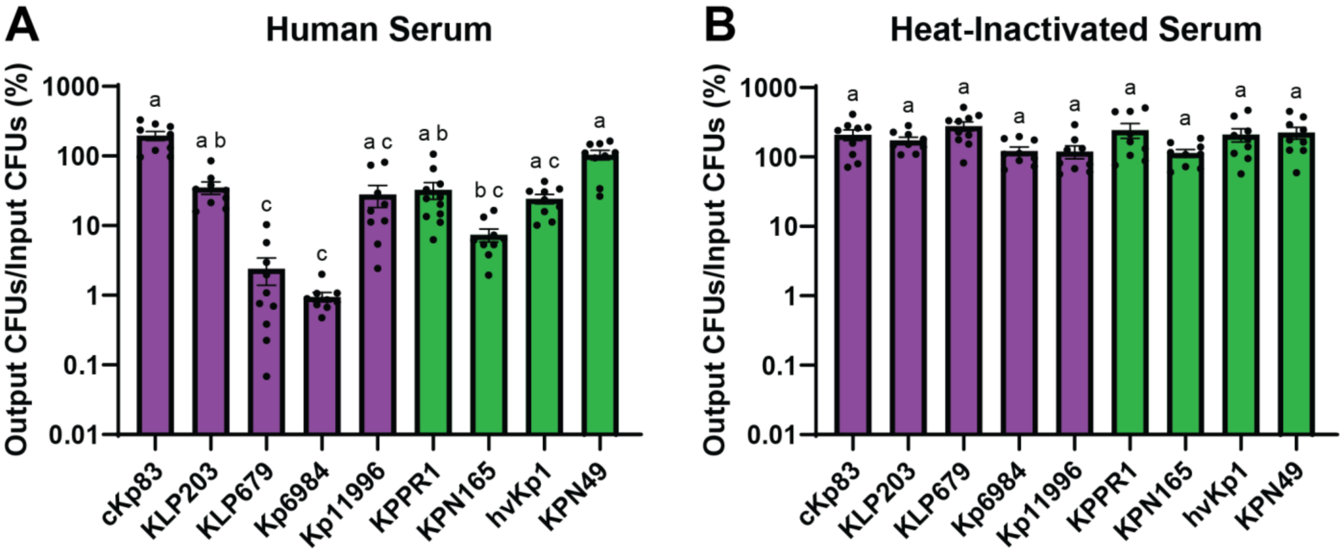

Figure S4. Human Serum Survival of Individual Strains

K2 cKp and hvKp were incubated in (**A**) 90% pooled human serum or (**B**) heat-inactivated serum for 90 minutes. Data are presented as the output CFUs divided by the input CFUs. Each bar identifies the mean, and error bars represent the standard error of the mean. To determine statistical significance, a Kruskal-Wallis test was used. Compact letter display was used to display multiple pairwise comparisons, where any two groups that share a letter have a p value greater than 0.05. Experiments were performed ≥ 3 independent times in triplicate.

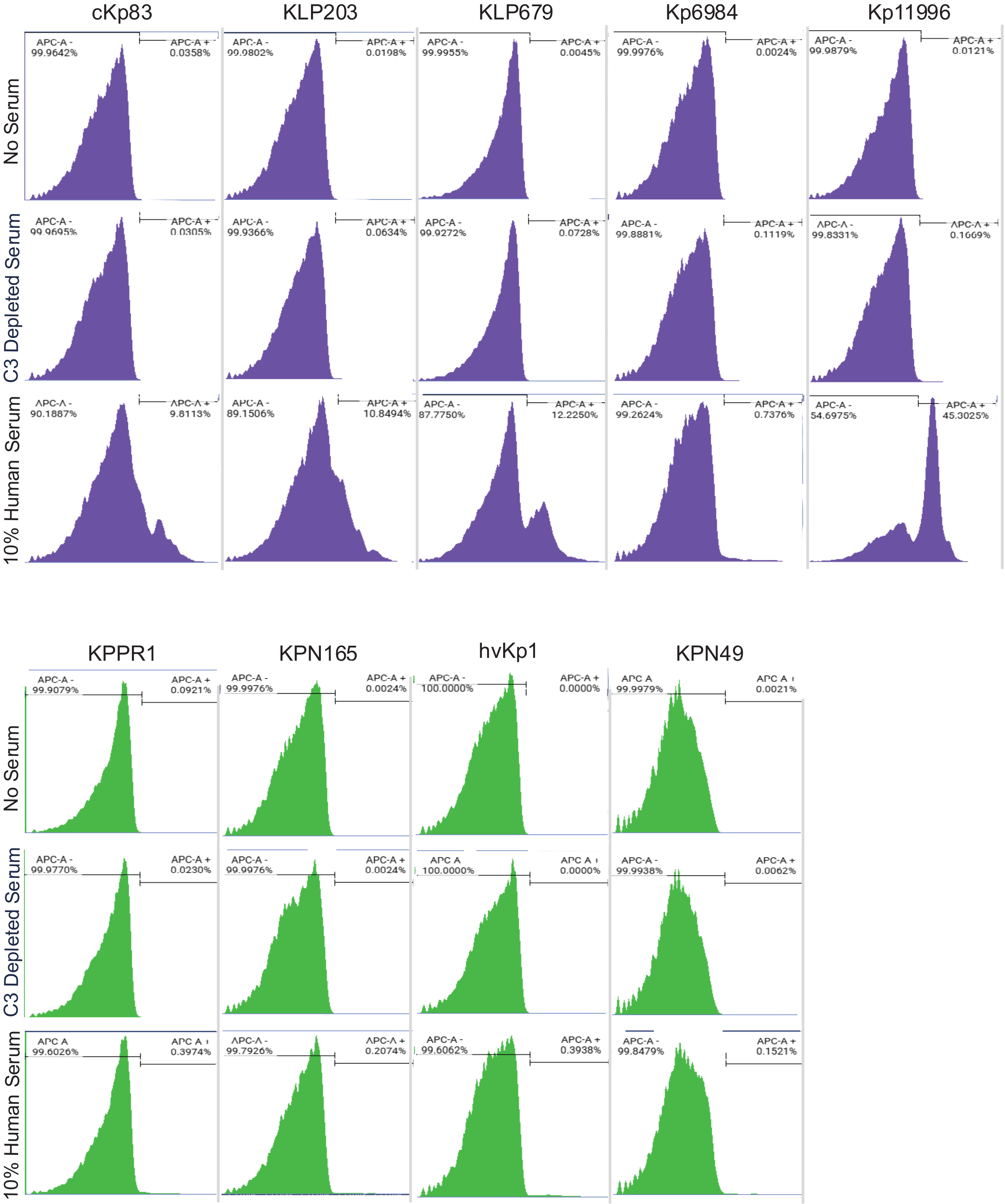

Figure S5. C3 Deposition of Individual Strains

K2 cKp and hvKp strains were incubated with 10% human serum for 30 minutes and stained with SYTO BC green nucleic acid stain and C3b/iC3b-APC. The SYTO BC positive population was selected and graphed as a histogram showing frequency of APC stain. The percent APC- or APC+ bacterial cells are reported above the histograms. Shown are representative histograms from experiments performed ≥ 2 independent times.

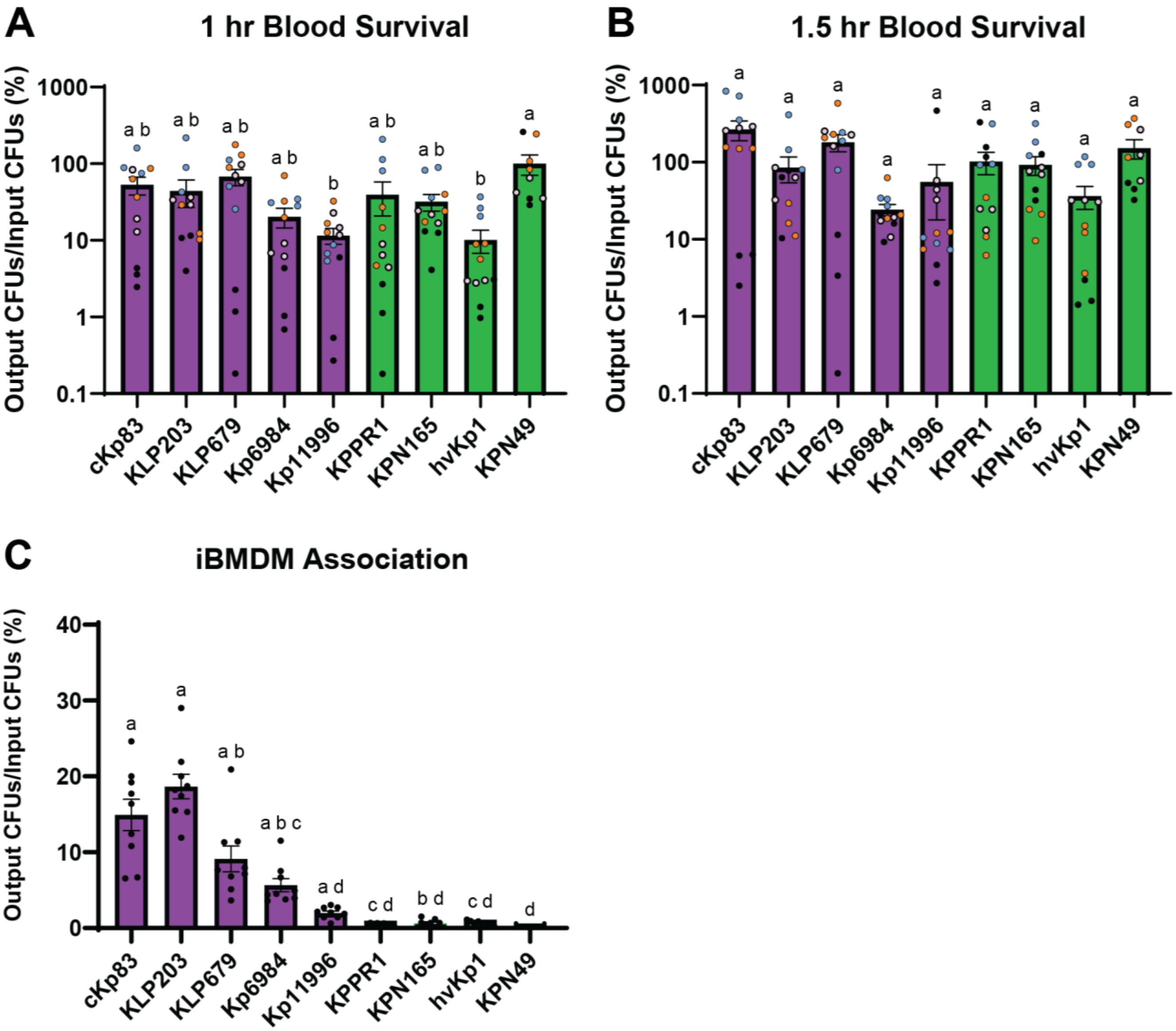

Figure S6. Blood Survival and iBMDM Association of Individual Strains

K2 cKp and hvKp were incubated in 90% fresh whole human blood for (**A**) 60 or (**B**) 90 minutes. Different colors identify data derived from each of the four unique donors. (**C**) Strains were incubated with immortalized bone-marrow derived macrophages (iBMDM) at an MOI of 10 for 2 hours, then washed prior to plating whole iBMDM lysates. For all graphs, data are presented as the percent output CFUs divided by the input CFUs. Each bar identifies the mean, and error bars represent the standard error of the mean. To determine statistical significance, a Kruskal-Wallis test was used. Compact letter display was used to display multiple pairwise comparisons, where any two groups that share a letter have a p value greater than 0.05. Experiments were performed ≥ 3 independent times in triplicate.

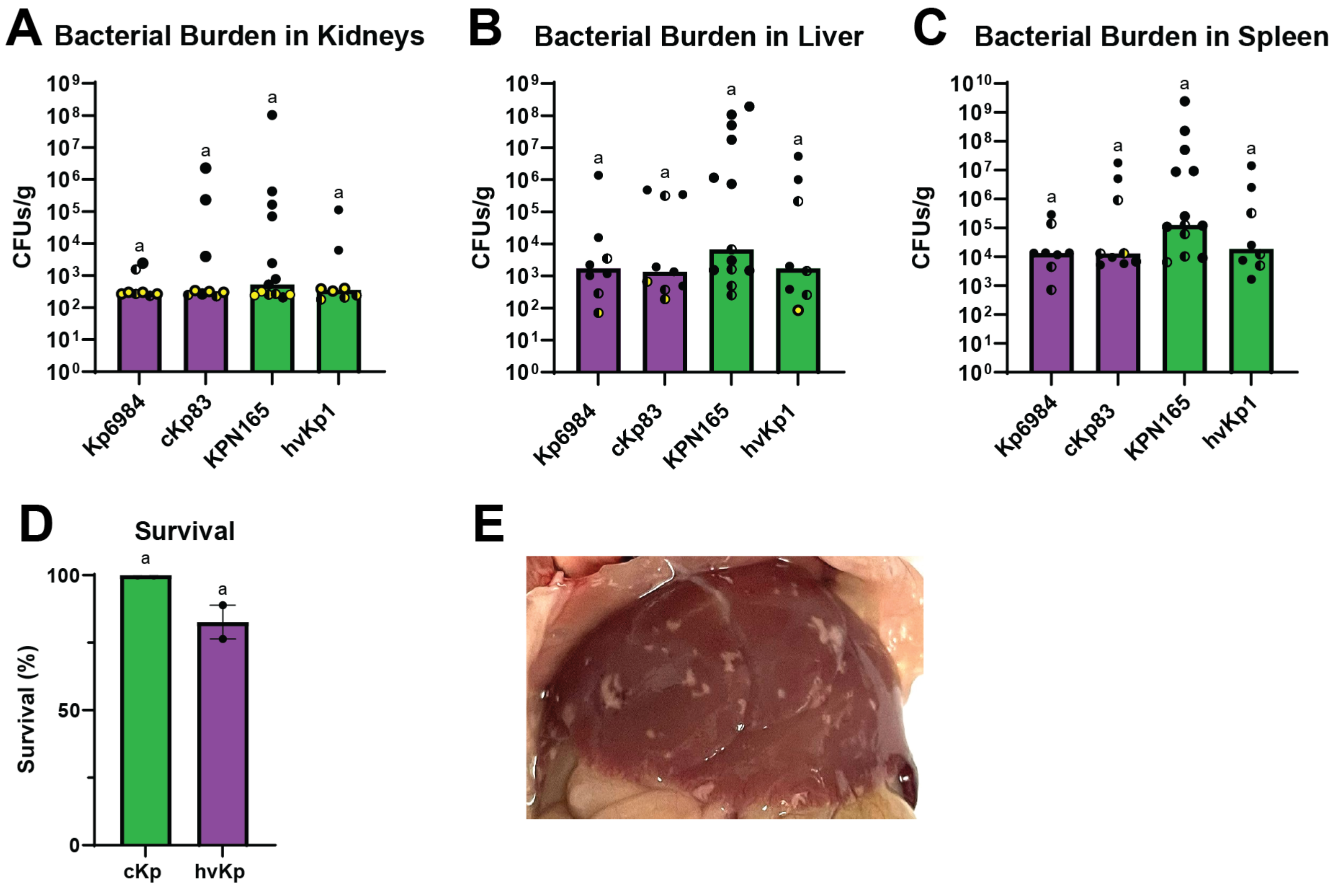

Figure S7. Bacterial Burdens of Individual Strains and Murine Survival

C57BL/6 mice were infected with 10^7 CFU of cKp (Kp6984 or cKp83) or hvKp (KPN165 or hvKp1) strains. After 24 hours, mice were humanely euthanized and bacterial burdens in (**A**) kidneys, (**B**) livers, and (**C**) spleens were enumerated. Bars represent the median and yellow dots represent points below the limit of detection. Half circles indicate male mice; full circles indicate female mice. (**D**) The percent of mice alive at 24 hours post-infection with K2 cKp or hvKp was plotted. (**E**) This is a representative image of an infected liver with inflammatory foci. Infections with each strain were performed ≥ 2 independent times with ≥ 8 total mice. To determine statistical significance in **A-C**, a Kruskal-Wallis test was used; a Mann-Whitney test was used to determine significance in **D**. Compact letter display was used to display multiple pairwise comparisons, where any two groups that share a letter have a p value greater than 0.05.
